# Supplementary material for: ATF4-dependent fructolysis fuels growth of glioblastoma multiforme
Source: Nat Commun. 2022 Oct 16;13:6108. doi: 10.1038/s41467-022-33859-9 (PMC9573865; doi:10.1038/s41467-022-33859-9)
Supplement: Supplementary file 1 — Supplementary Information [file 41467_2022_33859_MOESM1_ESM.pdf]

Supplementary Information

This PDF file includes:

Supplementary Fig. 1-6

Supplementary Table 1-3

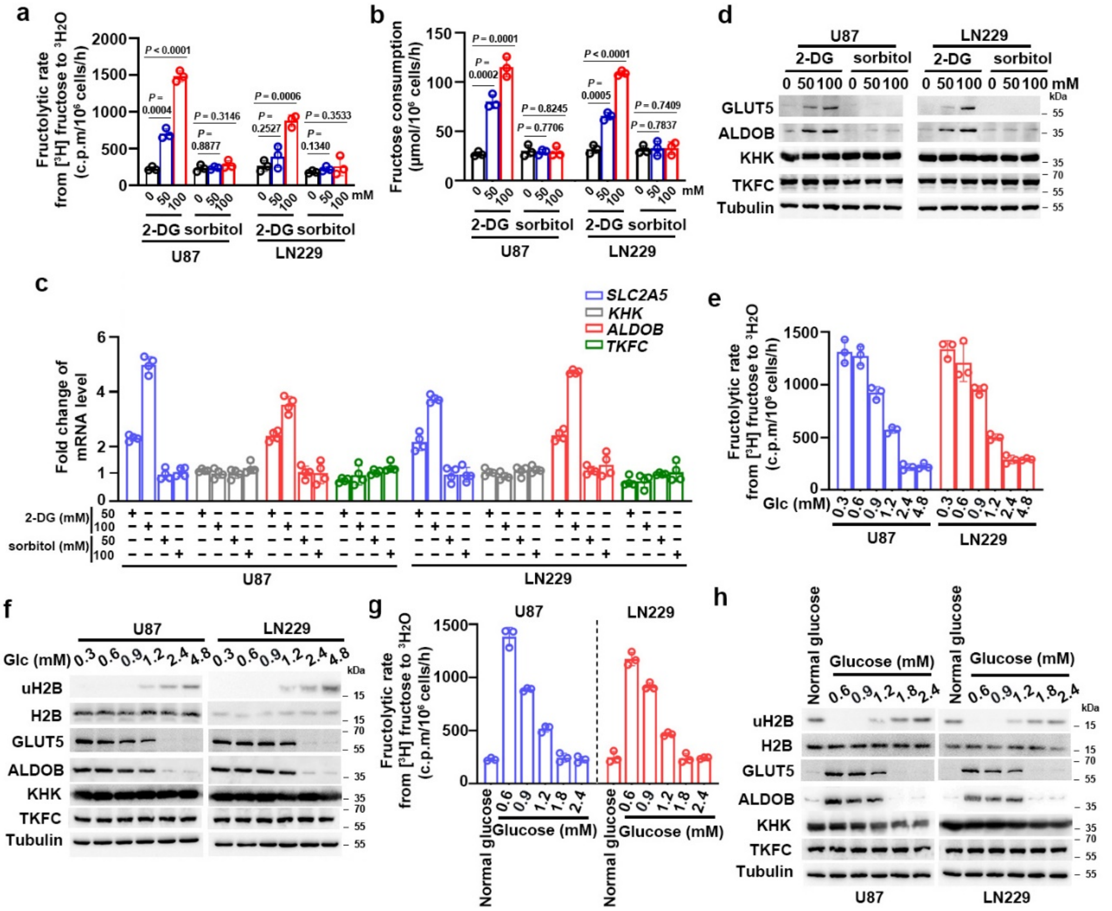

Supplementary Fig. 1: Energy stress promotes fructolysis. **a-d** U87 and LN229 cells cultured under high glucose condition (25 mM glucose) were treated with indicated concentrations of 2-DG or sorbitol for 18 hours. **a** Fructose metabolic rate was measured by monitoring the conversion of D-[5-^{3}H] fructose to ^3H_2O and normalized to cell number. c.p.m, counts per minute. **b** Fructose consumption was measured by incubating the cells with 10 mM of fructose for another 18 hours. **c**, **d** Indicated cells were analyzed by quantitative PCR (**c**) or immunoblotting using indicated antibodies (**d**). Data were presented as fold change caused by treatments with indicated concentrations of 2-DG or sorbitol (**c**). **e** U87 and LN229 cells were cultured with media

containing indicated concentrations of glucose for 12 hours. The fructose metabolic rate was measured by monitoring the conversion of D-[5-^{3}H] fructose to ^3H_2O and normalized to cell number. **f** U87 and LN229 cells cultured with media containing indicated concentrations of glucose for 12 hours were analyzed by immunoblotting with indicated antibodies. **g** U87 and LN229 cells cultured with media containing 0.6 mM glucose for 12 hours were treated with media containing indicated concentrations of glucose for another 12 hours. The fructose metabolic rate was measured by monitoring the conversion of D-[5-^{3}H] fructose to ^3H_2O and normalized to cell number. **h** U87 and LN229 cells cultured with media containing 0.6 mM glucose for 12 hours were treated with media containing indicated concentrations of glucose for another 12 hours, and then the cells were analyzed by immunoblotting with indicated antibodies. U87 and LN229 cells cultured under normal glucose (6 mM) condition served as controls. Data represent the mean ± SD of three (**a**, **b**, **e**, **g**) or four (**c**) independent experiments. The experiments were repeated three times independently with similar results (**d**, **f**, **h**). *P* values were determined by the one-way ANOVA (**a**, **b**). Source data are provided as a Source Data file.

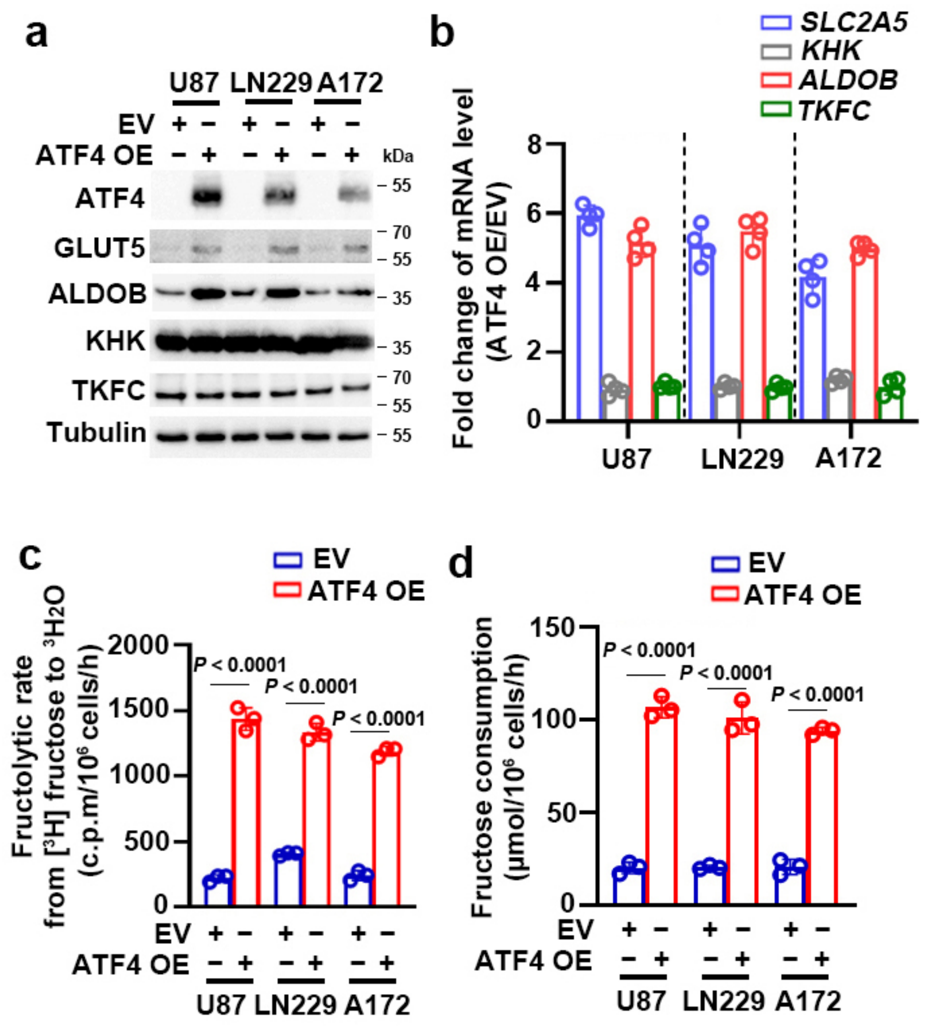

Supplementary Fig. 2: ATF4 activates fructolysis. **a**, **b** U87, LN229 and A172 cells without or with ATF4 overexpression (OE) were cultured under high glucose condition (25 mM glucose). The cells were analyzed by immunoblotting with indicated antibodies (**a**) and quantitative PCR (**b**). Data were normalized with β-actin mRNA levels and presented as the fold change of the mRNA levels of indicated genes in ATF4-overexpressing versus empty vector (EV)-expressing cells (**b**). **c** U87, LN229 and A172 cells without or with ATF4 OE were incubated with 5 mM fructose spiked with 10 μCi of D-[5-^{3}H] fructose for 1 hour. The fructose metabolic rate was measured by monitoring the conversion of D-[5-^{3}H] fructose to $^{3}H_2O$ and normalized to cell number. c.p.m, counts per minute. **d** U87, LN229 and A172 cells without or with of ATF4 OE

were cultured under high glucose condition (25 mM glucose) supplemented with 10 mM of fructose for 18 hours. The media were collected for analysis of fructose consumption. Data represent the mean ± SD of four (**b**) or three (**c**, **d**) independent experiments. The experiments were repeated three times independently with similar results (**a**). *P* values were determined by the two-tailed Student's *t*-test (**c**, **d**). Source data are provided as a Source Data file.

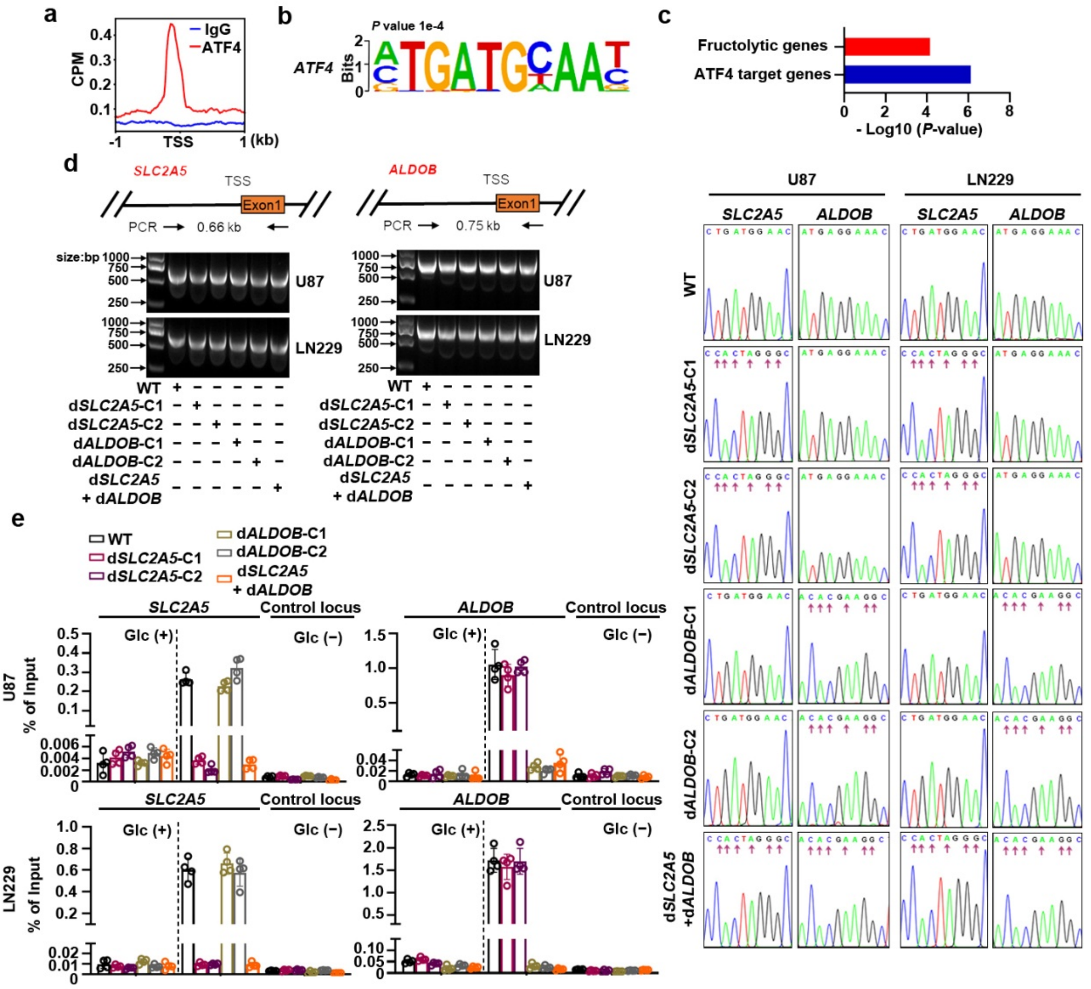

Supplementary Fig. 3: ATF4 binds to CARE motifs located within the promoters of fructolytic genes upon glucose deprivation. **a** U87 cells deprived of glucose for 18 hours were analyzed by ATF4 ChIP-seq analysis. Heatmaps of normalized read depth

within ± 1 kb of the TSSs of canonical chromosomes are shown. ChIP-seq with a normal IgG was served as a negative control. TSS, transcription start site; ChIP-seq, chromatin immunoprecipitation sequencing. CPM, counts per million. **b** The highest ranked *de novo* binding motif found within ± 50 bp of ATF4 ChIP peak positions. **c** The ATF4 ChIP peak-containing genes derived from U87 cells deprived of glucose for 18 hours were analyzed using the hypergeometric test to determine the enrichment of these genes to fructolysis (fructolytic genes) and ATF4 (ATF4 target genes) pathways. *P*-values obtained from the two-tailed hypergeometric test are indicated. **d** PCR products amplified from promoters of *SLC2A5* and *ALDOB* were separated on an agarose gel and sequenced. The mutated nucleotides located within CARE motifs are indicated by red arrows. CARE, C/EBP-ATF Response Element. **e** U87 and LN229 cells without or with disruption of the CARE motif located within the promoters of *SLC2A5* or *ALDOB* were treated without or with glucose deprivation for 18 hours. ChIP analyses of endogenous ATF4 binding to the loci of CARE motifs located within the promoters of *SLC2A5* or *ALDOB* were performed. ATF4 binding to a ~2 kb sequence downstream of the CARE motifs served as a control. The histogram shows the amount of immunoprecipitated DNA expressed as a percentage of the total input DNA. Data represent the mean ± SD of four independent experiments. Glc (+) and Glc (-) represent glucose-supplemented (25 mM glucose) and -deprived (1 mM glucose) condition, respectively (**e**). The experiment was performed twice independently with similar results (**a-d**). WT, wild type; d*SLC2A5*-C1/2 and d*ALDOB*-C1/2, cell clone 1/2 with disruption of the CARE motifs located within the promoters of *SLC2A5* and *ALDOB*,

respectively (**d**-**e**). Source data are provided as a Source Data file.

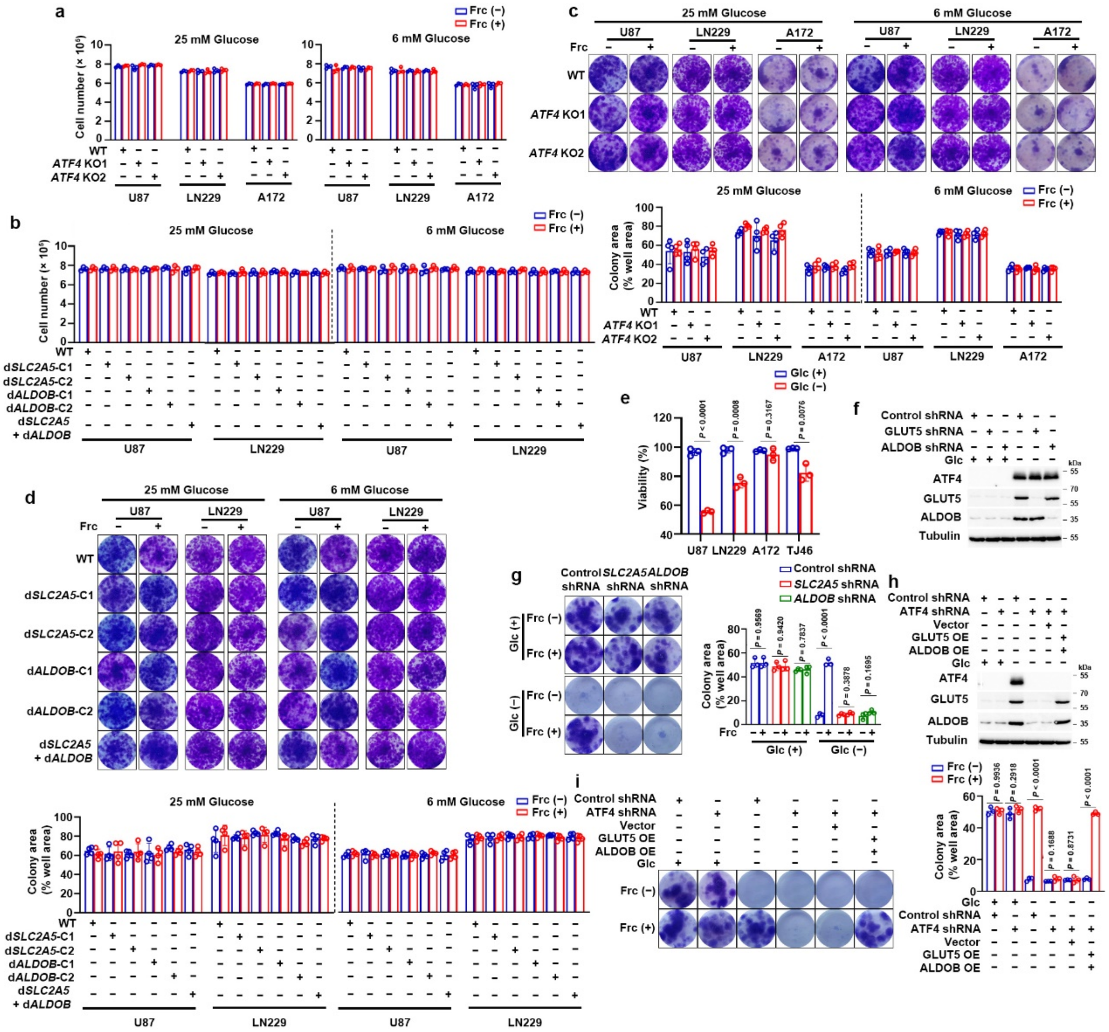

Supplementary Fig. 4: Fructose supplement does not affect glucose-induced proliferation and colony formation of GBM cells. **a-d** Indicated GBM cells without or with *ATF4* KO (**a**, **c**) and ATF4 binding-deficiency in the promoters of *SLC2A5* or *ALDOB* (**b**, **d**) were cultured under high glucose (25 mM) or normal glucose (6 mM) condition supplemented without or with 10 mM of fructose for 3 (**a**, **b**) or 14 (**c**, **d**) days. The cells were then collected and counted (**a**, **b**) or fixed by 4% paraformaldehyde and stained with crystal violet for colony formation analysis (**c**, **d**). **e** U87, LN229, TJ46 and A172 cells were treated without or with severe glucose deprivation (a treatment with 0

mM glucose) for 24 hours and stained with trypan blue (0.5%). The viable cells were counted. **f** U87 cells without or with knockdown *SLC2A5* or *ALDOB* were treated without or with glucose deprivation for 18 hours, and then analyzed by immunoblotting with indicated antibodies. **g** U87 cells without or with knockdown of *SLC2A5* or *ALDOB* were cultured under high glucose or glucose-deprived condition supplemented without or with 10 mM of fructose for 14 days. Colony formation analysis was performed. **h** U87 cells without or with *ATF4* knockdown and without or with overexpression (OE) of GLUT5 and ALDOB were treated without or with glucose deprivation for 18 hours, and then analyzed by immunoblotting with indicated antibodies. **i** U87 cells without or with *ATF4* knockdown and without or with OE of GLUT5 and ALDOB were cultured under high glucose or glucose-deprived condition supplemented without or with 10 mM of fructose for 14 days. Colony formation analysis was performed. Frc (-) and Frc (+) represent without and with 10 mM fructose supplementation, respectively (**a**-**d**, **g**, **i**). The experiments were repeated three times independently with similar results (**f**, **h**). The data are presented as the means ± SD from three independent experiments (**a**-**e**, **g**, **i**). *P* values were determined by the two-tailed Student's *t*-test (**e**, **g**, **i**). Source data are provided as a Source Data file.

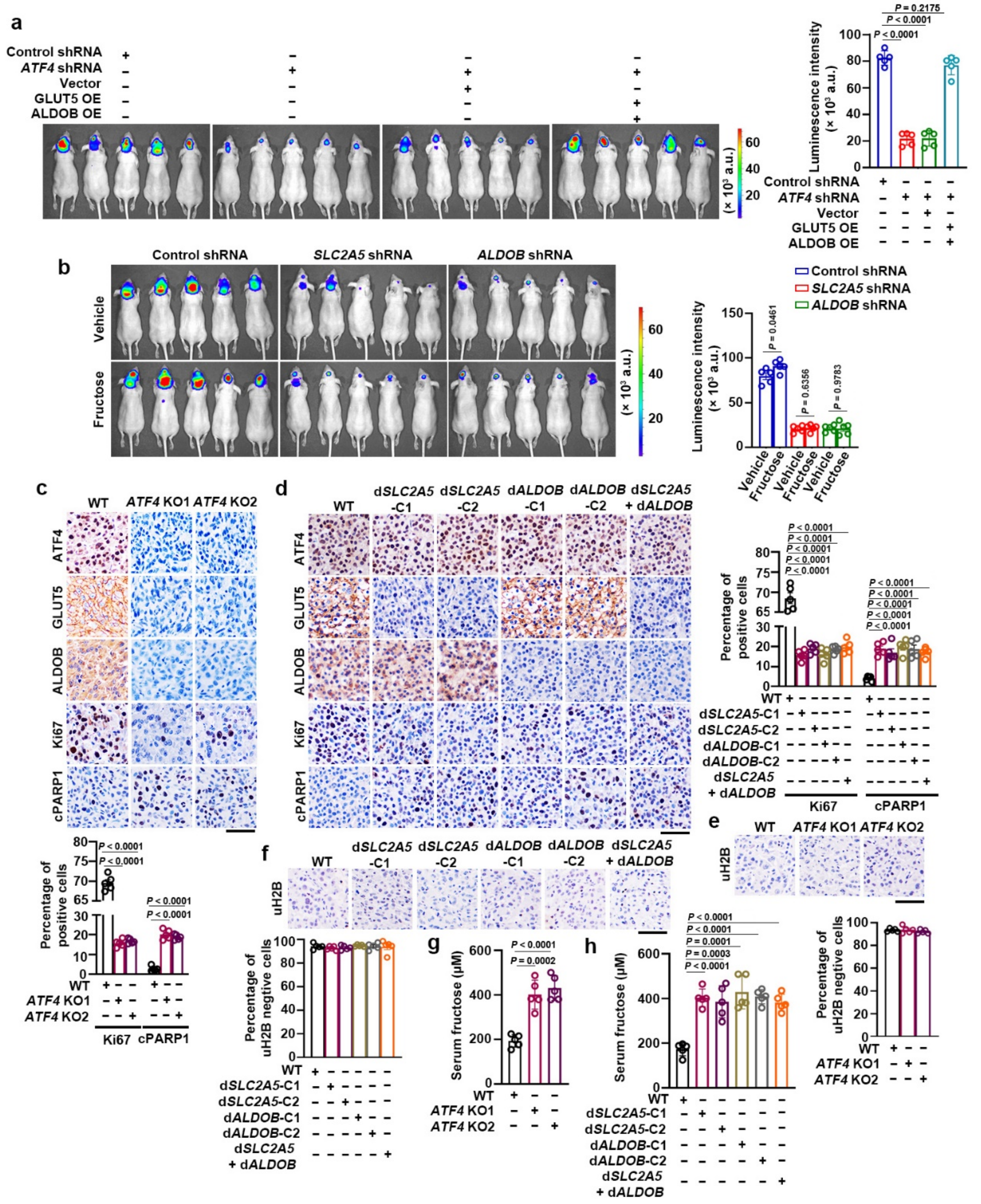

Supplementary Fig. 5: ATF4-dependent fructolysis supports GBM growth. **a** Luciferase-expressing U87 cells without or with *ATF4* knockdown and without or with overexpression (OE) of GLUT5 and ALDOB were intracranially injected into athymic nude mice (n = 5). Luminescence intensity derived from tumors was measured and relative luminescence intensity was shown. a.u., arbitrary unit. **b** Luciferase-expressing

U87 cells without or with knockdown of *SLC2A5* or *ALDOB* were intracranially injected into athymic nude mice (n = 5). Three days after tumor-cell injection, 200 μl of fructose (1 g kg^{-1}) or vehicle (PBS) was delivered to the mice via orogastrical administration daily for 18 days. Luminescence intensity derived from tumors was measured and relative luminescence intensity was shown. **c-f** Luciferase-expressing U87 cells without or with *ATF4* KO (**c** and **e**) or ATF4 binding-deficiency in the promoters of *SLC2A5* or *ALDOB* (**d** and **f**) were intracranially injected into athymic nude mice (n = 5). The mice were killed on day 21 post tumor-cell injection and sections of tumor tissues were immunohistochemically stained with indicated antibodies. Representative images are showed (**d** left; **c**, **e**, **f** top). Expression levels of indicated proteins were determined by the means of staining positive (**c** and **d**) or negative (**e** and **f**) rates quantified for ten microscopic fields of each tumor sample (**d** right; **c**, **e**, **f** bottom). cPARP1, cleaved PARP1. Scale bar, 50 μm. **g**, **h** Luciferase-expressing U87 cells without or with *ATF4* KO (**g**) or ATF4 binding-deficiency in the promoters of *SLC2A5* or *ALDOB* (**h**) were intracranially injected into athymic nude mice (n = 5). The tumor-bearing mice were fasted for 12 hours on day 21 post tumor-cell injection and serum fructose concentration was determined by a quantitative colorimetric assay. d*SLC2A5*-C1/2 and d*ALDOB*-C1/2, cell clone 1/2 with disruption of the CARE motifs located within the promoters of *SLC2A5* and *ALDOB*, respectively (**d, f, h**). Data represent the mean ± SEM of 5 mice (**a-f**, **g**, **h**). *P* values were determined by the one-way ANOVA (**a**, **c**, **d, g**, **h**) or two-tailed Student's *t*-test (**b**). Source data are provided as a Source Data file.

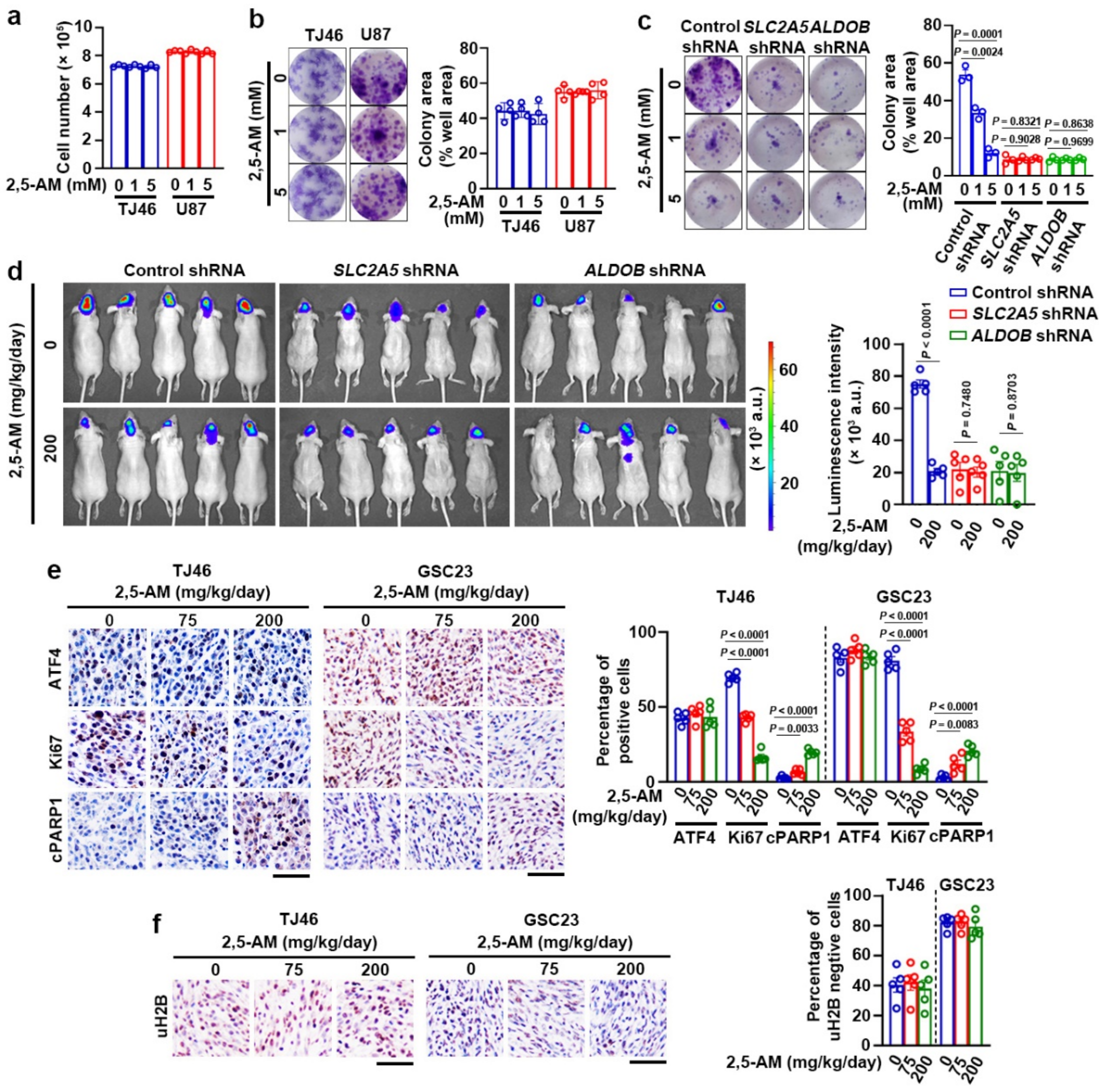

Supplementary Fig. 6: Pharmacological blockage of fructose utilization does not affect glucose-induced proliferation and colony formation of GBM cells *in vitro* and shows therapeutic potential *in vivo*. **a** TJ46 and U87 cells cultured under high glucose (25 mM) condition supplemented with 10 mM of fructose were treated with indicated concentrations of 2,5-AM for 3 days. The cells were then collected and counted. **b**, **c** TJ46 and U87 cells cultured under high glucose condition supplemented with 10 mM of fructose were treated with indicated concentrations of 2,5-AM for 14 days (**b**). U87 cells without or with knockdown of *SLC2A5* or *ALDOB* were cultured

under glucose-deprived condition supplemented with 10 mM of fructose in absence or presence of indicated concentrations of 2,5-AM for 14 days (**c**). Colony formation analysis was performed (**b**, **c**). **d** Luciferase-expressing U87 cells without or with knockdown of *SLC2A5* or *ALDOB* were intracranially injected into athymic nude mice (n = 5). Seven days after tumor-cell injection, 2,5-AM or vehicle (PBS) was delivered to the mice via intraperitoneal administration daily for 7 days. Luminescence intensity derived from tumors was measured and relative luminescence intensity was shown. a.u., arbitrary unit. **e**, **f** Luciferase-expressing TJ46 and GSC23 cells were intracranially injected into athymic nude mice (n = 5). Seven days after tumor-cell injection, indicated dosages of 2,5-AM were delivered to the mice via intraperitoneal administration daily. The mice were killed on day 14 (for TJ46 cells) or day 21 (for GSC23 cells) post tumor-cell injection and sections of tumor tissues were immunohistochemically stained with indicated antibodies. Representative images are showed (**e** and **f** left). Expression levels of indicated proteins were determined by the means of staining positive (**e**) or negative (**f**) rates quantified for ten microscopic fields of each tumor sample (**e** and **f** right). Scale bar, 50 μm. Data represent the mean ± SD of six (**a**), four (**b**) or three (**c**) independent experiments and the mean ± SEM of 5 mice (**d-f**). *P* values were determined by the one-way ANOVA (**c**, **e**) or two-tailed Student's *t*-test (**d**). Source data are provided as a Source Data file.

Supplementary Table 1. Genetic characterization of TJ46 cells

Genes	Genetic Alterations
EGFR-AS1	Fusion gene
EGFR	Copy number, n=4
BRAF	Copy number, n=3
VEGFA	Copy number, n=3
ALK	c.4587 C>G (D1529E); c.4472 A>G (K1491R); c.4381 A>G (I1461V)
PIK3CA	c.1173 A>G (I391M)
HER2(ERBB2)	c.3418 C>G (P1170A)

Supplementary Table 2. Multivariate analysis of overall survival in GBM patients (Cox's regression model)

Protein	Characteristics	HR	95% CI	*P* value
ATF4	Age, years (>49 vs ≤ 49)	2.233	0.895-5.570	0.085
	Sex (female vs male)	1.34	0.639-2.808	0.438
	Total resection (yes vs no)	0.573	0.254-1.292	0.179
	ATF4 staining scores (5-8 vs 0-4)	2.372	1.053-5.345	**0.037**
GLUT5	Age, years (>49 vs ≤49)	2.476	0.985-6.228	0.054
	Sex (female vs male)	1.38	0.659-2.890	0.393
	Total resection (yes vs no)	0.452	0.207-0.987	**0.046**
	GLUT5 staining scores (5-8 vs 0-4)	2.372	1.065-5.281	**0.035**
ALDOB	Age, years (>49 vs ≤49)	1.971	0.777-4.999	0.153
	Sex (female vs male)	1.406	0.671-2.948	0.367
	Total resection (yes vs no)	0.55	0.244-1.238	0.148
	ALDOB staining scores (5-8 vs 0-4)	2.581	1.176-5.664	**0.018**

Abbreviation: HR, hazard ratio; CI, confidence interval; PM, plasma membrane. *P* values were determined by the Cox regression model. Bold *P* values are statistically significant.

Supplementary Table 3. Primer and DNA oligonucleotide sequence information

ATF4 qPCR F	GTCCTCCACTCCAGATCATTC
ATF4 qPCR R	AGTCTGGCTTCCTATCTCCTTC
SLC2A5 qPCR F	GCAGAGTCGCCACATCATT
SLC2A5 qPCR R	CGTTGGAAGATACACCTGCA
ALDOB qPCR F	GCCACTCTCAACCTCAATGC
ALDOB qPCR R	GCCCGTCCATAAGAGAAACT
KHK qPCR F	GCTTGTATGGTCGTGTGAGG
KHK qPCR R	GGCATTGAAGGTGTCTCCA
TKFC qPCR F	AGCACTGGAGATGCCTGGCATT
TKFC qPCR R	ATGGAGACTGCAGCCACGTTAG
SLC2A5 promoter PCR F	ACAGTATTACACGGTGACTTGG
SLC2A5 promoter PCR R	CAACACAGAGTTCCCTCTGCA
ALDOB promoter PCR F	TGTTGGAATTGACTACTAATGAT
ALDOB promoter PCR R	CAAAGGCAAACCAATCTTCCCTTCTATA
PCDH ATF4 F	GATTCTAGAGCTAGCGAATTCATGACCGAAATGAGCTTCCTG
PCDH ATF4 R	CGCGGCCGCGGATCCCTACTTGTCGTCATCGTCTTTGTAGTCGGGGACCCTTTTCTTCC
pGreenFire1 *SLC2A5* F	ACAAAATTCAAAATTTTATCGATGAATTCGGTAGTGCACGCCTGTAATCCCAGCTAC
pGreenFire1 *SLC2A5* R	AGAGCCCGATTCACAGACTAGTTCTACCTCCCAACACAGAGTTCCCTCTGCAA
pGreenFire1 *ALDOB* F	ACAAAATTCAAAATTTTATCGATGAATTCGGAAATTCAAGGATTCAGAATCAATT
pGreenFire1 *ALDOB* R	AGAGCCCGATTCACAGACTAGTTCTACAAGCAGAGCCATCATGTTTTCAGCCA

pegRNA scaffold F	AGAGCTAGAAATAGCAAGTTAAAATAAGGCTAGTCCGTTATCAACTTGAAAAAGTGGCACCGAGTCG
pegRNA scaffold R	GCACCGACTCGGTGCCACTTTTTCAAGTTGATAACGGACTAGCCTTATTTTAACTTGCTATTTCTAG
SLC2A5 pegRNA spacer F	CACCGATACCGTAGACTGTTCCATCGTTTT
SLC2A5 pegRNA spacer R	CTCTAAAACGATGGAACAGTCTACGGTATC
SLC2A5 pegRNA 3'extension F	GTGCTTGTCCCACTAGGGCAGTCTACGGT
SLC2A5 pegRNA 3'extension R	AAAAACCGTAGACTGCCCTAGTGGGACAA
ALDOB pegRNA spacer F	CACCGACCCTCCATTTCACAGATGGTTTT
ALDOB pegRNA spacer R	CTCTAAAACCATCTGTGAAATGGAGGGTC
ALDOB pegRNA 3'extension F	GTGCGCCTTAGCCTTCGTGTCTGTGAAATGGAG
ALDOB pegRNA 3'extension R	AAAACTCCATTTCACAGACACGAAGGCTAAGGC
ATF4 sgRNA sequence-1	TCTCTTAGATGATTACCTGG
ATF4 sgRNA sequence-2	AGATGACCTTCTGACCACGT
ATF4 shRNA F	CCGGGCCTAGGTCTCTTAGATGATTCTCGAGAATCATCTAAGAGACCTAGGCTTTTTG
ATF4 shRNA R	AATTCAAAAAGCCTAGGTCTCTTAGATGATTCTCGAGAATCATCTAAGAGACCTAGGC
SLC2A5 shRNA F	CCGGCAGATCTTTGGTCTTCGGAATCTCGAGATTCCGAAGACCAAAGATCTGTTTTTG
SLC2A5 shRNA R	AATTCAAAAACAGATCTTTGGTCTTCGGAATCTCGAGATTCCGAAGACCAAAGATCTG
ALDOB shRNA F	CCGGCCTATTGTTGAACCAGAGGTATACTCGAGTATACCTCTGGTTCAACAATAGGTTTTTG
ALDOB shRNA R	AATTCAAAAACCTATTGTTGAACCAGAGGTATACTCGAGTATACCTCTGGTTCAACAATAGG

SLC2A5 ChIP qPCR F	TGCCATGCGTTCCTGACA
SLC2A5 ChIP qPCR R	GGTTTGGCTAAGTAAGTGGGTG
SLC2A5 ChIP control locus qPCR F	ACTGCAACCTCCACCTTCTG
SLC2A5 ChIP control locus qPCR R	GCACTTTGGGAGGCCGAG
ALDOB ChIP qPCR F	ACAGCTACACTAACATTCTTCCT
ALDOB ChIP qPCR R	TGTCTTTAGACCGTGGAGTTC
ALDOB ChIP control locus qPCR F	TGGAAGGCAGAGGCAAGA
ALDOB ChIP control locus qPCR R	TCAATCTTTCCACGTCAGCC
