# Supplementary material for: ATF4-dependent fructolysis fuels growth of glioblastoma multiforme
Source: Nat Commun. 2022 Oct 16;13:6108. doi: 10.1038/s41467-022-33859-9 (PMC9573865; doi:10.1038/s41467-022-33859-9)
Supplement: Supplementary file 3 — Reporting Summary [file 41467_2022_33859_MOESM3_ESM.pdf]

nature portfolio

Corresponding author(s): Xinjian Li

Last updated by author(s): Sep 19, 2022

Reporting Summary

Nature Portfolio wishes to improve the reproducibility of the work that we publish. This form provides structure for consistency and transparency in reporting. For further information on Nature Portfolio policies, see our Editorial Policies and the Editorial Policy Checklist.

Statistics

For all statistical analyses, confirm that the following items are present in the figure legend, table legend, main text, or Methods section.

n/a	Confirmed	
☐	☒	The exact sample size (n) for each experimental group/condition, given as a discrete number and unit of measurement
☐	☒	A statement on whether measurements were taken from distinct samples or whether the same sample was measured repeatedly
☐	☒	The statistical test(s) used AND whether they are one- or two-sided<br>*Only common tests should be described solely by name; describe more complex techniques in the Methods section.*
☐	☒	A description of all covariates tested
☒	☐	A description of any assumptions or corrections, such as tests of normality and adjustment for multiple comparisons
☐	☒	A full description of the statistical parameters including central tendency (e.g. means) or other basic estimates (e.g. regression coefficient) AND variation (e.g. standard deviation) or associated estimates of uncertainty (e.g. confidence intervals)
☐	☒	For null hypothesis testing, the test statistic (e.g. F, t, r) with confidence intervals, effect sizes, degrees of freedom and P value noted<br>*Give P values as exact values whenever suitable.*
☒	☐	For Bayesian analysis, information on the choice of priors and Markov chain Monte Carlo settings
☒	☐	For hierarchical and complex designs, identification of the appropriate level for tests and full reporting of outcomes
☐	☒	Estimates of effect sizes (e.g. Cohen's d, Pearson's r), indicating how they were calculated

Our web collection on statistics for biologists contains articles on many of the points above.

Software and code

Policy information about availability of computer code

Data collection	Bioluminescent imaging of mice was recorded using an IVIS Lumina System coupled with the Living Image data-acquisition software program version 4.7.3. Raw Illumina sequencing output was converted to fastq format using FASTQC (v0.11.9). Immunoblots were visualized by a ChemiScope 6000 Exp instrument.
Data analysis	The intensity of immunoblotting bands was quantified by the Image Lab software program version 6.1. IBM SPSS Statistics version: 23.0 was used to perform the two-tailed Spearman's coefficient correlation analysis, the multivariate analysis, the two-tailed log-rank tests, and the two-tailed Student t-tests. The two-tailed fisher's exact test and the two-tailed hypergeometric test were performed by using the R statistical program (R Core Team, 2013). The peak photon flux within a region of mouse was recorded and quantified using an IVIS Lumina System coupled with the Living Image data-acquisition software program version 4.7.3. The softwares used for ChIP-seq data analyses were described in the Methodology session of ChIP-seq. Trim Galore program (v0.6.7) was used to remove adapters and low-quality bases in the sequencing reads. BWA software (v0.7.9a) and SAMtools package (v0.1.19) were used for sequence alignments. Single-nucleotide polymorphisms (SNPs) were identified with the GATK 3.8 scripts. PCR duplicates were marked by the Picard script (v1.115) and re-aligned to the local sequences around indels using the GATK 3.8 scripts to reduce false-positive detection of SNPs. The copy number variations (CNVs) were detected by the Control-FREEC 11.5 package and further annotated by the ANNOVAR program (v20200608). Colony formation areas in each dish were analyzed by ImageJ software.

For manuscripts utilizing custom algorithms or software that are central to the research but not yet described in published literature, software must be made available to editors and reviewers. We strongly encourage code deposition in a community repository (e.g. GitHub). See the Nature Portfolio guidelines for submitting code & software for further information.

Data

Policy information about availability of data

All manuscripts must include a data availability statement. This statement should provide the following information, where applicable:

- Accession codes, unique identifiers, or web links for publicly available datasets
- A description of any restrictions on data availability
- For clinical datasets or third party data, please ensure that the statement adheres to our policy

The ChIP-seq data (https://www.ncbi.nlm.nih.gov/geo/query/acc.cgi?acc=GSE188633) and TJ46 cell whole-genome sequencing data (https://www.ncbi.nlm.nih.gov/geo/query/acc.cgi?acc=GSE202644) that support the findings of this study have been deposited in the Gene Expression Omnibus. The data supporting the findings of this study are available within the Article and its Supplementary Information file. Source data are provided with this paper.

Field-specific reporting

Please select the one below that is the best fit for your research. If you are not sure, read the appropriate sections before making your selection.

[x] Life sciences [ ] Behavioural & social sciences [ ] Ecological, evolutionary & environmental sciences

For a reference copy of the document with all sections, see nature.com/documents/nr-reporting-summary-flat.pdf

Life sciences study design

All studies must disclose on these points even when the disclosure is negative.

Sample size	We used online tools available at http://www.biomath.info/power/ttest.htm to determine the sample or group sizes of the experiments.
Data exclusions	No data were excluded from analysis.
Replication	All the presented experimental results were performed at least three times independently. All attempts at replication were successful.
Randomization	The samples for each experiment were randomized to be examined (No specific methods were used).
Blinding	No blinding was performed due to none of the analyses reported involved procedures that could be influenced by investigator bias.

Reporting for specific materials, systems and methods

We require information from authors about some types of materials, experimental systems and methods used in many studies. Here, indicate whether each material, system or method listed is relevant to your study. If you are not sure if a list item applies to your research, read the appropriate section before selecting a response.

Materials & experimental systems

n/a	Involved in the study	
[ ]	[x]	Antibodies
[ ]	[x]	Eukaryotic cell lines
[x]	[ ]	Palaeontology and archaeology
[ ]	[x]	Animals and other organisms
[ ]	[x]	Human research participants
[x]	[ ]	Clinical data
[x]	[ ]	Dual use research of concern

Methods

n/a	Involved in the study	
[ ]	[x]	ChIP-seq
[x]	[ ]	Flow cytometry
[x]	[ ]	MRI-based neuroimaging

Antibodies

Antibodies used

Mouse monoclonal antibody recognizing GLUT5 (clone name: E-2, sc-271055, lot: #L0214, 1:1000 for immunoblotting) was purchased from Santa Cruz Biotechnology.
Rabbit polyclonal antibody recognizing KHK (#HPA007040, lot: CH4164, 1:1000 for immunoblotting) was purchased from Sigma.
Rabbit polyclonal antibody recognizing ALDOB (#18065-1-AP, lot: 00046063, 1:1000 for immunoblotting and 1:100 for immunohistochemical staining) was obtained from Proteintech.
Rabbit polyclonal antibody recognizing TKFC (#A15421, 1:400 for immunoblotting) was purchased from ABclonal.
Rabbit monoclonal antibodies recognizing Ki67 (clone name: EPR3610, #ab92742, lot: GR220263-2, 1:5000 for immunohistochemical staining) was purchased from Abcam.
Mouse monoclonal antibody recognizing tubulin (clone name: B-5-1-2, sc-23948, lot: #F2118, 1:1000 for immunoblotting) was purchased from Santa Cruz Biotechnology.

Rabbit monoclonal antibody recognizing ATF4 (clone name: D4B8, #11815, lot: 5, 1:100 for ChIP and 1:1000 for immunoblotting) was purchased from Cell Signaling Technology.
Rabbit monoclonal antibody recognizing phospho-elF2α (Ser51) (clone name: D9G8, #3398, lot: 6, 1:1000 for immunoblotting) was purchased from Cell Signaling Technology.
Rabbit monoclonal antibody recognizing elF2α (clone name: D7D3, #5324, lot: 9, 1:1000 for immunoblotting) was purchased from Cell Signaling Technology.
Rabbit monoclonal antibody recognizing phospho-ACC (Ser79) (clone name: D7D11, #11818, lot: 4, 1:1000 for immunoblotting) was purchased from Cell Signaling Technology.
Rabbit monoclonal antibody recognizing ACC (clone name: C83B10, #3676, lot: 12, 1:1000 for immunoblotting) was purchased from Cell Signaling Technology.
Rabbit monoclonal antibodies recognizing cleaved PARP (clone name: D64E10, #5625, lot: 13, 1:100 for immunohistochemical staining) was purchased from Cell Signaling Technology.
Ubiquityl-Histone H2B (Lys 120) (clone name: D11, #5546, lot: 7, 1:1000 for immunoblotting and 1:100 for immunohistochemical staining) was purchased from Cell Signaling Technology.
Histone H2B (clone name: D2H6, #12364, lot: 3, 1:1000 for immunoblotting) was purchased from Cell Signaling Technology.
Rabbit polyclonal antibody recognizing GLUT5 (#PA580023, lot: 3371AA42, 1:1000 for immunohistochemical staining) were obtained from Thermo Fisher Scientific.
Rabbit monoclonal antibodies recognizing ATF4 (clone name: EPR18111, #ab184909, lot: GR3360041-1, 1:100 for immunohistochemical staining) were obtained from Abcam.
Mouse monoclonal antibodies recognizing Flag tag (clone name: M2, #F3165, 1:2000 for immunoblotting) was purchased from Sigma.
Horseradish peroxidase-conjugated goat anti-mouse (#G-21040, 1:5000 for immunoblotting) secondary antibodies was purchased from Thermo Fisher Scientific.
Horseradish peroxidase-conjugated goat anti-rabbit (#G-21234, 1:5000 for immunoblotting) secondary antibodies was purchased from Thermo Fisher Scientific.

Validation

In general, we relied on data provided by the manufacturer's for validation as well as references in publications.
Mouse monoclonal antibody recognizing GLUT5 (#sc-271055) for WB, IP, IF, IHC-P, ELISA in mouse, rat, human. https://www.scbt.com/p/glut5-antibody-e-2/.
Rabbit polyclonal antibody recognizing KHK (#HPA007040) for ICC, IHC,WB in human. https://www.sigmaaldrich.cn/CN/zh/product/sigma/hpa007040.
Rabbit polyclonal antibody recognizing ALDOB (#18065-1-AP) for WB, IP, IHC, IF,ELISA in Human, Mouse, Rat. https://www.thermofisher.cn/cn/zh/antibody/product/ALDOB-Antibody-Polyclonal/18065-1-AP.
Rabbit polyclonal antibody recognizing TKFC (#A15421) for WB in Human, Mouse, Rat. https://www.citeab.com/antibodies/6030145-a15421-tkfc-rabbit-pab.
Rabbit monoclonal antibodies recognizing Ki67 (#ab92742) for Flow Cyt (Intra), WB, IHC-P, ICC in human. https://www.abcam.cn/Ki67-antibody-EPR3610-ab92742.html.
Mouse monoclonal antibody recognizing tubulin (sc-23948) for WB, IHC, IP in human, mouse. https://www.scbt.com/p/alpha-tubulin-antibody-b-5-1-2/.
Rabbit monoclonal antibody recognizing ATF4 (#11815) for WB, IP, IF, ChIP in Human, Mouse, Rat. https://www.cellsignal.cn/products/primary-antibodies/atf-4-d4b8-rabbit-mab/11815.
Rabbit monoclonal antibody recognizing phospho-elF2α (Ser51) (#3398) for WB, IP, IHC in Human, Mouse, Rat, monkey, D. melanogaster. https://www.cellsignal.com/products/primary-antibodies/phospho-eif2a-ser51-d9g8-xp-rabbit-mab/3398.
Rabbit monoclonal antibody recognizing elF2α (#5324) for WB, IP, IHC in Human, Mouse, Rat, Monkey. https://www.cellsignal.cn/products/primary-antibodies/eif2a-d7d3-xp-rabbit-mab/5324.
Rabbit monoclonal antibody recognizing phospho-ACC (Ser79) (#11818) for WB, IP, IHC, IF in Human, Mouse, Rat. https://www.cellsignal.com/products/primary-antibodies/phospho-acetyl-coa-carboxylase-ser79-d7d11-rabbit-mab/11818.
Rabbit monoclonal antibody recognizing ACC (#3676) for WB, IP, IHC, IF in Human, Mouse, Rat, Hamster. https://www.cellsignal.com/products/primary-antibodies/acetyl-coa-carboxylase-c83b10-rabbit-mab/3676.
Rabbit monoclonal antibodies recognizing cleaved PARP (#5625) for WB, IP, IHC, IF in Human, Monkey. https://www.cellsignal.com/products/primary-antibodies/cleaved-parp-asp214-d64e10-xp-rabbit-mab/5625.
Rabbit polyclonal antibody recognizing GLUT5 (#PA580023) for WB, IHC, IF, Flow Cyt in Human, Rat. https://www.thermofisher.cn/cn/zh/antibody/product/GLUT5-Antibody-Polyclonal/PA5-80023.
Rabbit monoclonal antibodies recognizing ATF4 (#ab184909) for Flow Cyt (Intra), WB, IHC-P, ICC/IF, IP in Human. https://www.abcam.cn/atf-4-antibody-epr18111-ab184909.html.
Mouse monoclonal antibodies recognizing Flag tag (clone name: M2, #F3165) for WB, IP. https://www.sigmaaldrich.cn/CN/zh/product/sigma/f3165.
Rabbit monoclonal antibodies recognizing Ubiquityl-Histone H2B (Lys 120) (clone name: D11, #5546, lot: 7) for WB, IHC. https://www.cellsignal.cn/products/primary-antibodies/ubiquityl-histone-h2b-lys120-d11-xp-rabbit-mab/5546.
Rabbit monoclonal antibodies recognizing Histone H2B (clone name: D2H6, #12364, lot: 3) for WB, IHC. https://www.cellsignal.cn/products/primary-antibodies/histone-h2b-d2h6-rabbit-mab/12364.

Eukaryotic cell lines

Policy information about cell lines

Cell line source(s)	Human GBM cell lines including U87 (#HTB-14), LN229 (#CRL-2611), A172 (#CRL-1620) were obtained from ATCC. The TJ46 cells derived from a GBM primary tissue were used in our previous studies (PMID: 30279734, PMID: 34620861). GSC23 cell line was a gift from the Department of Neuro-Oncology, The University of Texas MD Anderson Cancer Center.
Authentication	All cell lines used in this study were authenticated with STR profiling.
Mycoplasma contamination	All cell lines used in this study were negative for the tests of mycoplasma contamination.
Commonly misidentified lines (See ICLAC register)	No cell lines used in this study were found in the database of commonly misidentified cell lines maintained by ICLAC and NCBI Biosample.

Animals and other organisms

Policy information about studies involving animals; ARRIVE guidelines recommended for reporting animal research

Laboratory animals	The 6-week-old Balb/c male athymic nude mice were purchased from GemPharmatech (Nanjing, China). Mice were housed in a pathogen-free environment with the temperature maintained at 23 ± 2°C and relative humidity at 50 to 65% under a 12 h/12 h light/dark cycle with free access to food and water.
Wild animals	No wild animals were used.
Field-collected samples	No field-collected samples were used.
Ethics oversight	The animals were treated in accordance with the Guide for the Care and Use of Laboratory Animals published by the National Academy of Sciences and the National Institutes of Health. The use of animals in this study was approved by the Institutional Animal Care and Use Committee of the Center for Animal Experiments of the Institute of Biophysics, Chinese Academy of Sciences.

Note that full information on the approval of the study protocol must also be provided in the manuscript.

Human research participants

Policy information about studies involving human research participants

Population characteristics	Information about the patient sex, age, surgery date, overall survival time, and resection status are given in the source data. There were 37 males and 31 females, aged 32-80 years, with GBM. All patients had received standard clinical treatments.
Recruitment	The samples were collected randomly after surgical operation.
Ethics oversight	The use of patient specimens and the relevant database was approved by the Human Research Ethics Committee of the First Affiliated Hospital of Nanjing Medical University. The use of PDX cells derived from a GBM primary tissue was approved by the Human Research Ethics Committee of the Tianjin Medical University.

Note that full information on the approval of the study protocol must also be provided in the manuscript.

ChIP-seq

Data deposition

☒ Confirm that both raw and final processed data have been deposited in a public database such as GEO.

☒ Confirm that you have deposited or provided access to graph files (e.g. BED files) for the called peaks.

Data access links *May remain private before publication.*	https://www.ncbi.nlm.nih.gov/geo/query/acc.cgi?acc=GSE188633
Files in database submission	GBM Input<br>GBM IgG<br>GBM TF-Glc<br>GBM Input rep<br>GBM IgG rep<br>GBM TF-Glc rep<br>GBM Glc(+) Input<br>GBM Glc(+) IgG<br>GBM Glc(+) TF<br>GBM Glc(+) Input rep<br>GBM Glc(+) IgG rep<br>GBM Glc(+) TF rep

Genome browser session
(e.g. UCSC)

https://asia.ensembl.org/Homo_sapiens/Info/Index?db=core

Methodology

Replicates

The ChIP-seq experiments were performed twice independently with similar results.

Sequencing depth

The median sequencing depth ChIP-Seq Samples were 40 million, the raw read number for each are below:
Sample_Name, Raw_Reads.
GBM_Input, 42344608
GBM_IgG, 41603562
GBM_TF-Glc, 43828448
GBM_Input_rep, 60731086
GBM_IgG_rep, 40603556
GBM_TF-Glc_rep, 57785960
GBM_Glc(+)_Input, 40688136
GBM_Glc(+)_IgG 47143960
GBM_Glc(+)_TF, 41429786
GBM_Glc(+)_Input_rep, 40303024
GBM_Glc(+)_IgG_rep, 42236874
GBM_Glc(+)_TF_rep, 41374870

Antibodies

Rabbit monoclonal antibodies against ATF4 (clone name: D4B8, #11815, lot: 5) was purchased from Cell Signaling Technology.

Peak calling parameters

The peaks were called from alignment results using call peak function of MACS2 (v2.2.7.1) with default parameters and annotated by the annotatePeaks.pl function of HOMER (v4.8.3) using GENCODE19 as reference.

Data quality

The annotated peaks were filtered using the following criteria: each peak must have the ''TSS'' annotation and locates within the canonical chromosomes with false discovery rate (FDR) ≤ 0.1. The rest peaks were further filtered according to their distances (± 1 kb) to the TSS.

Software

Sequencing reads were qualified using FASTQC (v0.11.9) followed by adapter removal and reserving the reads with length at least 36 bases using Trim Galore (v0.6.7). The processed reads were aligned to GRCh38 (hg38) reference genome using BWA (v0.7.9a) followed by duplicate reads removal using sambamba (v0.8.0).
