# Supplementary material for: ATF4-dependent fructolysis fuels growth of glioblastoma multiforme
Source: Nat Commun. 2022 Oct 16;13:6108. doi: 10.1038/s41467-022-33859-9 (PMC9573865; doi:10.1038/s41467-022-33859-9)
Supplement: Supplementary file 4 — Description of Additional Supplementary Files [file 41467_2022_33859_MOESM4_ESM.docx]

**Description of Additional Supplementary Files**

File Name: Supplementary Data 1

Description: ATF4 ChIP-seq peaks detected within ± 1 kb of the transcription start sites of canonical chromosomes
