# Supplementary material for: ATF4-dependent fructolysis fuels growth of glioblastoma multiforme
Source: Nat Commun. 2022 Oct 16;13:6108. doi: 10.1038/s41467-022-33859-9 (PMC9573865; doi:10.1038/s41467-022-33859-9)

Figure 1d

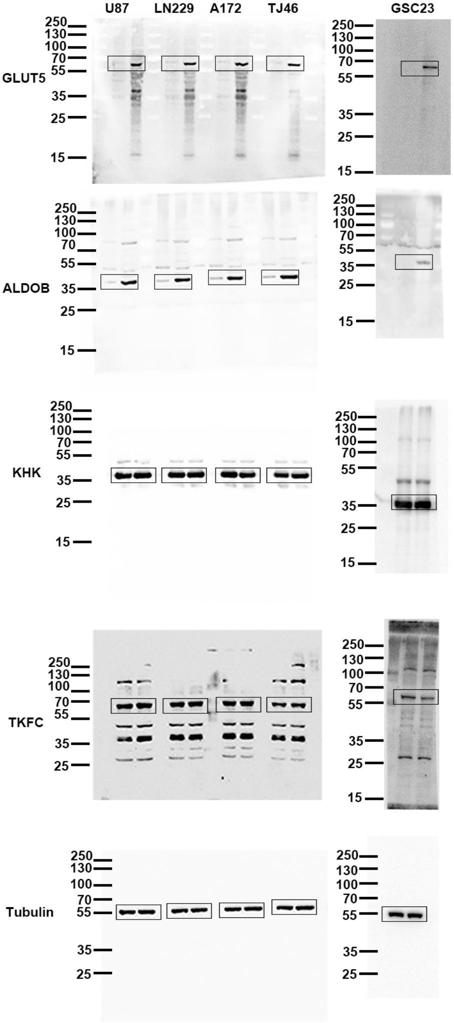

U87
LN229
A172
TJ46
GSC23
GLUT5
ALDOB
KHK
TKFC
Tubulin
250
130
100
70
55
35
25
15

Figure 2b

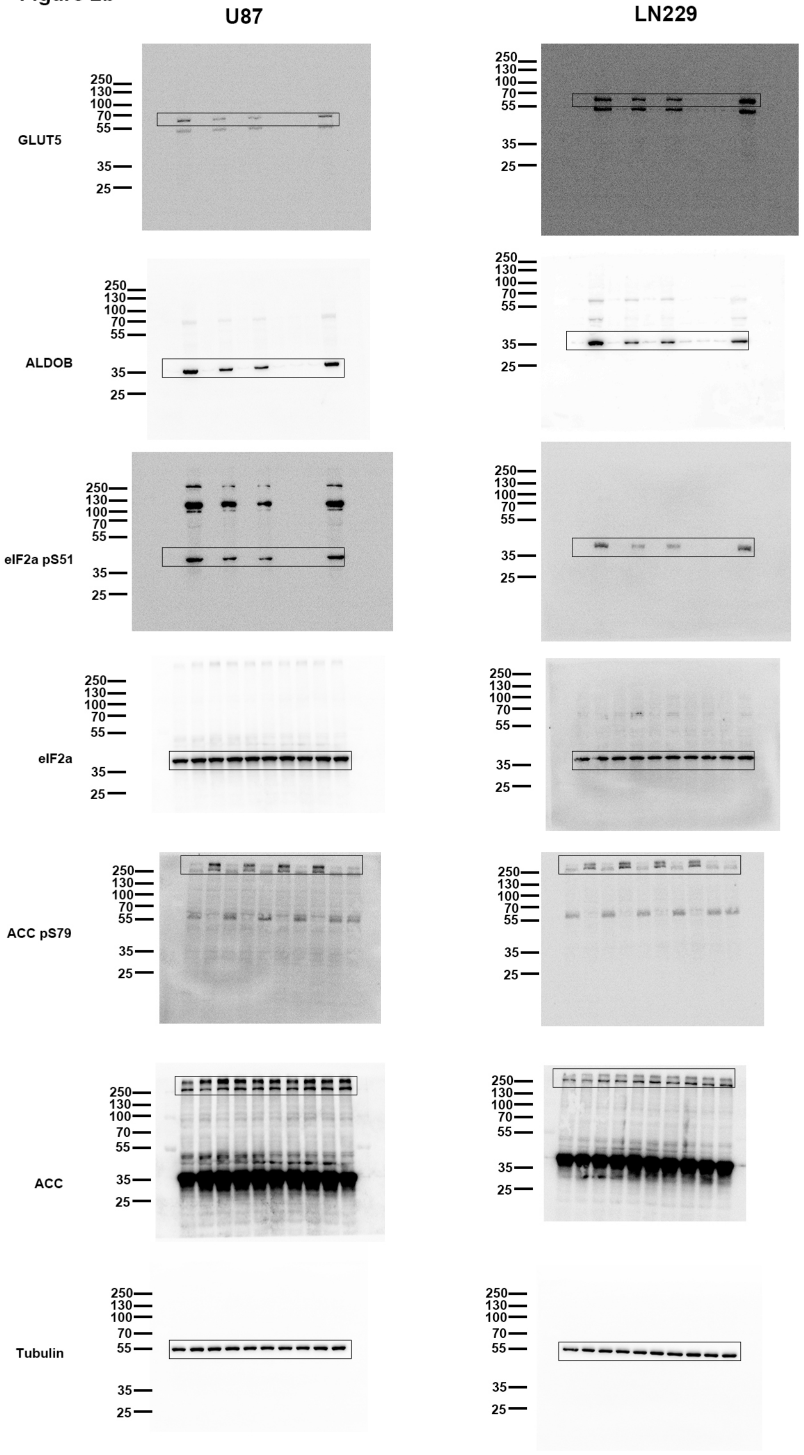
U87
LN229
GLUT5
ALDOB
eIF2a pS51
eIF2a
ACC pS79
ACC
Tubulin
250
130
100
70
55
35
25

Figure 2c

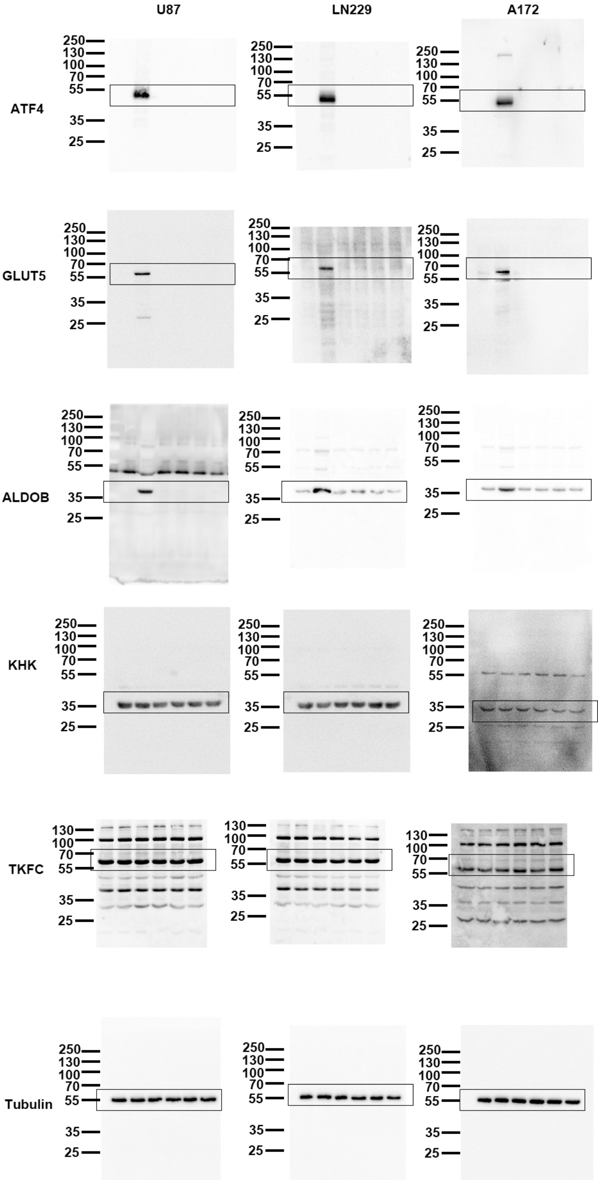
U87
LN229
A172
ATF4
GLUT5
ALDOB
KHK
TKFC
Tubulin
250
130
100
70
55
35
25

Figure 3d

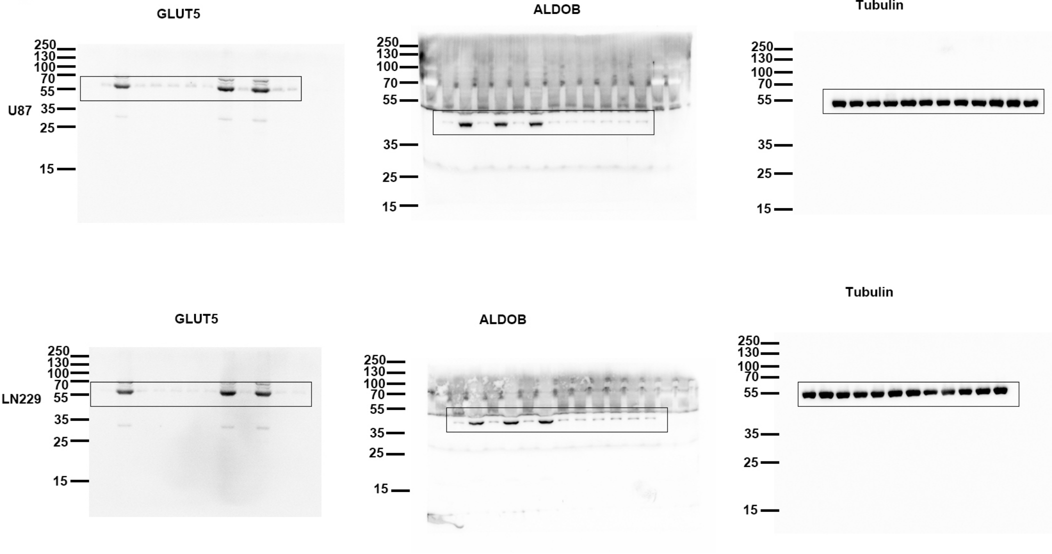
GLUT5
ALDOB
Tubulin
U87
250
130
100
70
55
35
25
15
GLUT5
ALDOB
Tubulin
LN229

Supplementary Fig.1d

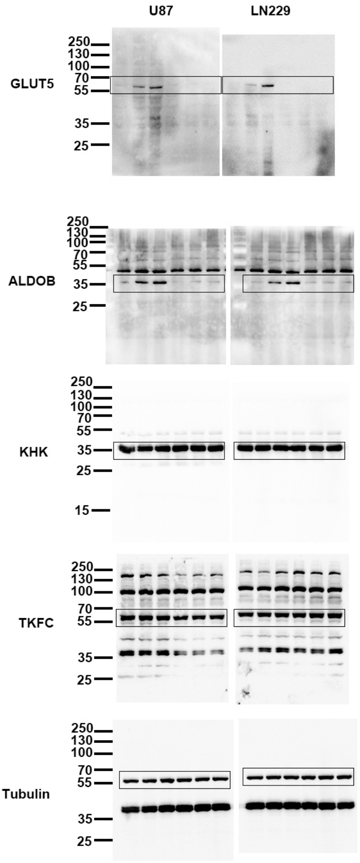

Supplementary Fig.1f

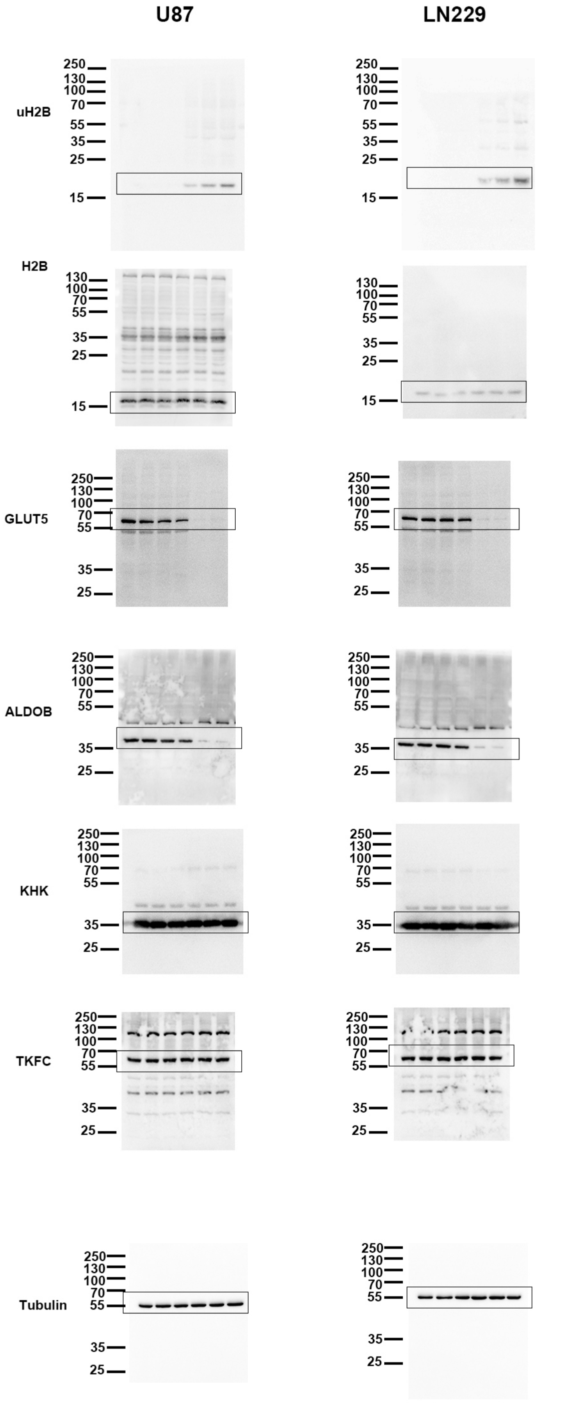

Supplementary Fig.1h

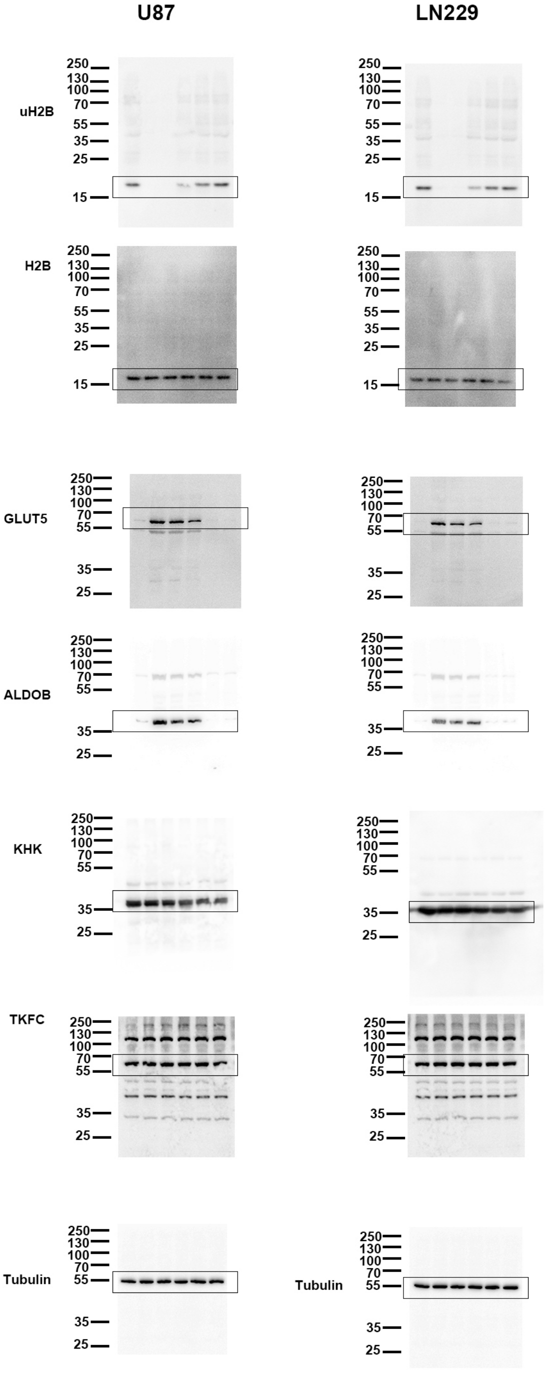

Supplementary Fig.2a

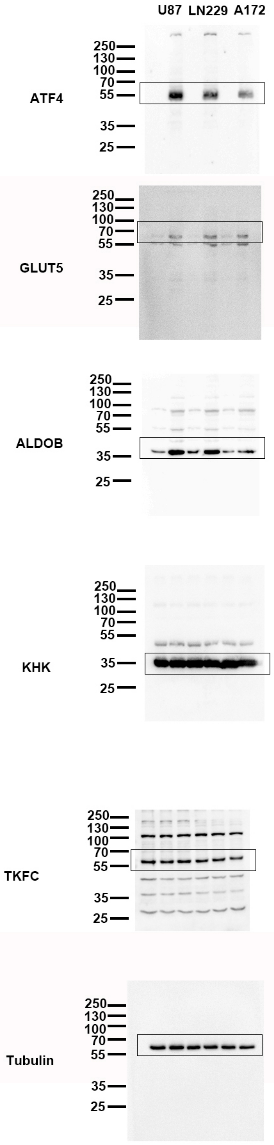

Supplementary Fig.4f Supplementary Fig.1h

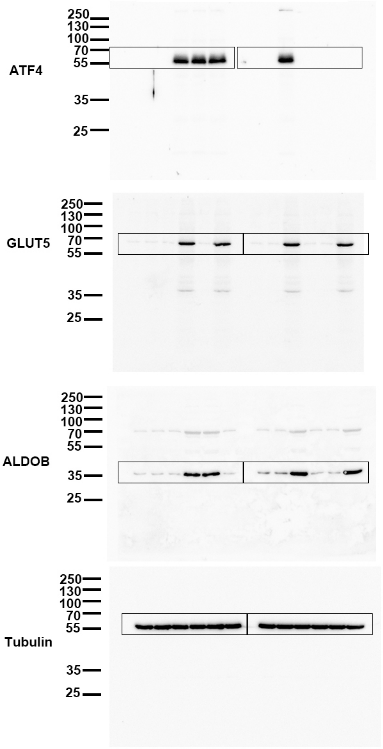

Supplement: Supplementary file 6 — Source Data [file 41467_2022_33859_MOESM6_ESM.zip › Source data WB.pdf]
